# The Colombian Healthy Child Contest: in search of Latin America’s “ideal child” in the 1930s

**DOI:** 10.1590/S0104-59702023000100005en

**Published:** 2023-04-03

**Authors:** Iván Darío Olaya Peláez

**Affiliations:** i Temporary lecturer/researcher, Faculté des Affaires Internationales/Université Le Havre. Le Havre – Normandie – France iolaya@gmail.com

**Keywords:** healthy child contest, Colombia, child protection, racial improvement, eugenics, concurso niño sano, Colombia, protección infantil, mejoramiento racial, eugenesia

## Abstract

This article analyzes healthy child contests as a medical and socio-political strategy implemented in Latin America to protect childhood, thus ensuring the future of the “race” and the nation. These contests blended degeneration, racial theories, and state interventionism and gained momentum in the 1930s with the rise of eugenics. This article examines the contest in Colombia, which was implemented under the Liberal Republic (1930-1946); even though this competition was defined by its national context, a broader international perspective improves understanding. Questions are also raised about the efforts of the Liberal government to strengthen the idea of national identity through education and health programs.

## Resumen

Este artículo analiza los concursos de niño sano como una estrategia médica y sociopolítica implementada en América Latina para proteger a la niñez, asegurando así el futuro de la “raza” y de la nación. Estos concursos combinaron la degeneración, las teorías raciales y el intervencionismo estatal y cobraron impulso en la década de 1930 con el auge de la eugenesia. Este artículo examina la contienda en Colombia, que se implementó bajo la República Liberal (1930-1946); aunque esta competencia fue definida por su contexto nacional, una perspectiva internacional más amplia mejora la comprensión. También se plantean preguntas sobre los esfuerzos del gobierno liberal para fortalecer la idea de identidad nacional a través de programas de educación y salud.

On June 22, 1937 the Colombian government enacted Decree 1201 reinstating the national Healthy Child Contest (HCC) on the grounds that “2) the future of the race relies mostly on childhood protection so that our future men can develop and grow up in the best hygienic, biological, and social conditions; … 4) parents’ efforts to preserve their children’s health, not only those that are sick or retarded, but all, should be encouraged” (Decreto n.1201…, 1 Sept. 1937). Four months later, the Colombian newspaper *El Tiempo* published a photo of the two winners of the contest, which was held in the capital city of Bogotá. Leopoldo Méndez (named the healthiest child in the Chapinero nursery) and Betulia Toledo (the healthiest baby served by the La Goutte de Lait Organization) appeared alongside Bogotá Mayor Manuel Antonio Rueda Vargas, Doctor Ignacio Moreno Pérez (Director of the Municipal Department of Hygiene) and a “selected group of respected ladies of the city.” The contest, presided over by Colombian First Lady María Michelsen de López, had taken place days before at the presidential palace.

Contests rewarding healthy children were part of a national and international “crusade” to protect children that embodied the future of the nation and the “race” by rewarding Colombian mothers who ensured proper physical, psychological and moral development of their babies. The first contests were held in the early twentieth century in Latin America, when licensed physicians started to focus on high child mortality (Birn, 2007, p.684-685); they gained traction in the 1930s alongside the rise of eugenics on the continent. This international scientific, social, and political movement to “improve the race” by regulating individual sexuality and reproduction focused on protecting children – the figure at the heart of this movement in Latin America.

This article analyzes the HCC in Colombia as one of the medical and socio-political strategies that combined eugenic and sociobiological principles, degeneration, racial theories, and state interventionism by the Liberal Republic (1930-1946) to make the country a “civilized” nation. Despite the its popularity of the contest across Latin America, this program that strengthened the image of the “ideal baby” as one of the main elements in the construction of the nation-state has not yet been studied in detail. As a transnational strategy, it was widely discussed and advised in several pan-American congresses and conferences such as the Pan American Sanitary Conferences, the Pan American Child Congresses, and the Pan American Conferences on Eugenics and Homiculture.

In investigating the HCC in Colombia, this article draws on Birn’s (2007, p.687) statement that child health “became a central component of the modernizing agenda that would last over several generations” in Latin America. It also raises questions about the efforts of the Liberal government to strengthen the idea of national identity through political programs centered on education and health. We also argue that even though a socio-political strategy prevailed under the Liberal governments in the 1930s, the eugenic narrative stressing the biological function of the population to modernize and civilize the nation persisted. As Catalina Muñoz Rojas (2022, p.2) points out, the reforms proposed by the “Liberal Republic” were founded on the idea that the lower classes were “essentialized … and in need of assistance, and in a state of precariousness and helplessness.” Our analysis initially examines the origins of this contest as a medico-social strategy to reduce child mortality by promoting the principles of puericulture as well as how the event spread across Latin America. Next, we analyze its implementation in Colombia as a strategy to “preserve the race” and strengthen national identity through child protection. Documentary sources include three decrees enacted in the late 1930s and early 1940s related to the creation of the HCC, some newspaper articles published in Colombia’s *El Tiempo* on the contest held on October 12, 1937, and a report written by Doctor Benjamín Otálora, Vice-Director of the Second Division of the National Department of Hygiene.

## A medico-social strategy to fight child mortality and “preserve the race”

The modern notion of childhood as a social category distinct from adulthood with its own characteristics began to take shape in the seventeenth and eighteenth centuries. The concept of “infant grace” that emerged during this period transformed childhood into something that needed to be protected and educated (Peyre, 1960, p.487). Throughout the nineteenth century, childhood became a “social problem” as society became concerned about the welfare of children and the government started protective interventions that included regulating children’s industrial work and improving school instruction and hygiene conditions (Armengaud, 1973, p.303). In this way, childhood shifted from a family issue to a social one under the purview of the state. Child mortality, which had previously been seen as normal, became a social problem as a result of this new perspective (see Morel, 2004).

The high rates of infant and child mortality in Western Europe and Latin America in the late nineteenth century demanded the implementation of socio-medical programs in response. Even though high mortality had been systematic throughout the century, the new view of childhood and the engagement of the medical community (who took on a “national mission” to protect children’s lives in terms of quality as well as quantity) gave this problem a collective dimension rather than simply the sum of individual cases (Olaya, 2020, p.121).

Puericulture – the “art of cultivating children,” a concept coined by the French physician Alfred Caron in 1865 and later popularized worldwide by the French doctor Adolphe Pinard (1844-1934) – was undoubtedly one of the most popular medico-social programs that not only reaffirmed the triumph of medicine and public health over child mortality as avoidable (Morel, 2001, p.45), but also allowed doctors to justify their interventions in neonatal care, thus establishing their authority over babies even prior to birth. The medicalization of childhood also meant that medical experts had power over the bodies of pregnant women, since the health of humanity and future generations relied on them (Quiroz, 2015, p.182). As Pinard (1908, p.6) himself defined it, puericulture is the “science that searches for knowledge related to the reproduction, preservation, and improvement of the human species.”

To this end, Pinard published a manual entitled *La Puériculture du premier âge* in 1904 teaching mothers how to protect their babies from harmful external influences so they could be clean, avoid extreme temperatures, reduce the risk of falls or trauma, how to feed them so they would not die of malnourishment, how to look after them if they fell ill, and to avoid diseases by keeping vaccinations up-to-date (Pinard, 1938, p.XI). Following these precepts reduced the chances that a baby could die. If this did happen, the mother was to blame for lacking responsibility and devotion to her child: a “responsible mother” would never let her children die (on the role of woman as the “mother of the nation,” see Miranda, 2020).

The rise of public health, a medical discipline that focused on prevention and collective health as essential to the development of the nation, and the proliferation of puericulture led to the emergence of contests like the HCC.

The first baby contest was held in 1880 in Paris, organized by the Mutual Aid Society in partnership with the city council to spread a social, hygienist message and fight child mortality. The winners received prizes that included medals, certificates, and sustainable development savings accounts (Un Concours…, s.d.).^1^

The notion of contests to select healthy children did not take long to spread throughout the western world along with other programs to protect childhood and fight child mortality. Thanks to the establishment of transnational scientific networks and the multiplication of international meetings of experts interested in child welfare (mainly doctors and hygienists, later including jurists, education specialists, anthropologists, and sociologists), transnational social knowledge was developed on this new social category and medico-social programs like these contests were organized in different countries across the Americas. The initial contests were held mainly in large cities in countries including Cuba, Mexico, and Venezuela as early as the 1910s (Olaya, 2020, p.128). Cuba, a regional leader in institutionalizing puericulture with the foundation of the Division of Child Hygiene in 1913, organized the first contests; in 1912 the physician Enrique Núñez de Villavicencio (1872-1916), founder of the Division of Child Hygiene, started Baby Exhibitions and Motherhood Prize Programs. These contests were part of a national political and scientific strategy to reduce child mortality, illegal abortion, and divorce (García González, Álvarez Peláez, 2007, p.41).^2^

Although child mortality was the original motivation for the HCC, the circulation of population degeneration theories, associated with race and poverty and the surge of eugenics in the 1920s and 1930s, gave the contest new impetus. Indeed, interest in the physical, physiological, and mental dimensions of childhood increased when racial degeneration theories ramped up during the early twentieth century and designated child morbidity and mortality as signs of this alleged degeneration. Eugenics elevated childhood as a social category to a higher level and consequently made it an object of public policies. Because physical and mental “defects” were likely to be transmitted from one generation to the next, doctors and hygienists focused their attention on the welfare of children even prior to procreation. Childhood was considered the stage in human evolution that could be most easily shaped by biology because of the “malleability” present during this phase (Sáenz Obregón, Saldarriaga, Ospina, 1997a, p.185). From this point, consequently, quantity and “quality” were important in the crusade for child protection, with competitions like the HCC as proof.

In a region dominated by a neo-Lamarckian, preventive interpretation of eugenics (in other words, the hereditary transmission of acquired characteristics, despite some nuances and emphases) (Armus, 2016, p.151), along with the Catholic Church’s power over social and political issues, puericulture became a discipline through which Latin American experts channeled less coercive and more persuasive eugenic ideas (Birn, 2007, p.690). Maternity and child protection comprised the main thrust of the eugenics movement in Latin America thanks to the prestige of puericulture among gynecologists, obstetricians, and pediatricians in this region (see Miranda, 2020, p.141-52; Reggiani, 2019, p.27). Nationalist discourses that proliferated during the first half of the twentieth century (most notably in the 1930s) reinforced the idea that childhood required complete attention in order to produce a vigorous population of “responsible” and “normal” citizens (Stern, 2016, p.8).

The idea of “preserving the race” through child protection solidified with each congress on children as eugenic discourse strengthened throughout the 1920s and the 1930s. For instance, the Fifth Pan American Child Congress, which took place in Havana in 1927, advised governments in the Americas to “bear in mind that the future of the human species, with regards to its existence and ultimate progress, demands not to prevent individuals from procreating and to provide the child with the greatest pains both in terms of education and positive applications of the medical science” (Actas…, 1928, p.53).

Unsurprisingly, Cuba was the first Latin American country to incorporate eugenic ideas into its health child competition. Not only was it among the first nations to focus its efforts on child protection, as mentioned above, but it was also the first to institutionalize eugenics with the foundation of the Homiculture League in 1910 (Schell, 2010, p.480).^3^ Within this scope, the Baby Exhibitions and Motherhood Prizes programs were transformed into the Motherhood, Homiculture, and Eugenic Fertility Contest in the 1920s. This competition was held until 1936, with a broader age range for participating children and rewards for marriages that produced more than ten children. According to the Comptroller to the Nation, Doctor Aurelio Méndez, the contest would save millions of lives from “physical and mental degenerations” as it encouraged mothers to learn how to raise their children, thus arming them with physiological factors to help defend them from morbidity (García González, Álvarez Peláez, Naranjo Orovio, 1999, p.313).

The child contests also incorporated a racial slant. Anne Shelby Blum noted that even though the description of the Healthiest Baby Contest in Mexico suggested that the entire population was eligible and that the criteria for evaluating health might have been transparent, the judges favored babies with European features (Blum, 2009, p.142). The racial element was closely intertwined with socioeconomic class. Local authorities targeted poor urban families who managed to raise healthy children in spite of their socioeconomic status, breastfeeding them and incorporating the lessons in puericulture taught at various local health departments. When the Mexican newspaper *El Universal* published an article announcing the winners of the local Healthy Working-Class Baby Contest held on April 27, 1923, it specified that the first prize winner was 6-month-old Agustín Arellano, whose parents “lived in extreme poverty.” His mother had “followed the rules given at the Child Hygiene Center” and produced a “charming” child with no signs of disease (La vez…*,* 24 Apr. 2021).

Targeting the low and working classes seemed natural, since poverty and this population’s “deplorable conditions” could put the social system at risk (González Leandri, 2015, p.382). Although these social classes were victims of disproportionate urbanization and ruthless capitalism, they were considered to cause their own misery due to their depraved morals and vicious social behaviors (alcoholism, prostitution, and parents’ incapacity to raise and look after their children) (Fassin, 24 Aug. 2020). Hereditary theories and eugenics which “scientifically” proved that these social diseases could be passed from one generation to the next urged even more political and scientific circles to focus their medico-social programs on this population, especially their children. Since they were the future of the nation, it was essential to stop them from acquiring “racial poisons” (Stepan, 1991, p.17, 101).^4^

The contests for healthy children seem to have been well received across Latin America, especially in the 1930s. In fact, these events were praised by the Puerto Rican hygienist Aristide A. Moll during the 10th Pan American Sanitary Conference, held in Bogotá in 1938. In his speech, the editor-in-chief of the *Pan American Journal of Public Health* mentioned all the child welfare programs carried out by the nations in the region to reduce child mortality, including the HCC. He listed the countries where these events were organized as part of a national plan to produce “healthier generations for the benefit of the respective countries:” Venezuela, Cuba, Mexico, as well as Brazil and Peru, where they were held within the framework of “Child Week” (Moll, 1939, p.897). Colombia was also mentioned, as the country had organized its first national HCC in 1937 to reward mothers whose children were both physically and mentally healthy, as the “future of the race relied heavily on child protection” (Decreto n.1201…, 1 Sept. 1937).

## The Liberal Republic in the “national crusade” for child protection and national identity

The organization of the HCC in Colombia in the mid-1930s was one strategy implemented by the Liberal government, which in taking over the executive branch of the government in 1930 after almost 50 years of Conservatism, centered its program on public hygiene and education, most notably under President Alfonso López Pumarejo (1934-1938). The administration focused on public hygiene and education in order to “regenerate the population” and lift it out of its “cultural savagery” and “backwardness.” This socio-political approach that made state intervention in social matters more essential did not replace the “medical strategy;” this strategy, which predominated during the two previous decades, sought to “reinvigorate the nation’s biological tissue as the population was seen in terms of race” (Sáenz Obregón, Saldarriaga, Ospina, 1997b, p.267; Muñoz Rojas, 2022, p.92).

From that time on, national unity relied on citizens who were to be shaped in the interest of a national identity. According to this perspective, the educational and cultural levels of the citizens would lay the groundwork to construct the “civilized” nation-state (Díaz, 2008, p.49). This framework reasserted the value of the child as the essential foundation of the nation-state. As such, children needed to be defended and protected, since the progress of the “race” and, in turn, the homeland, relied on them.

As part of this campaign in favor of child protection, the Liberal government founded and restructured the Child Welfare Section, run by the National Department of Hygiene (NDH), in 1931 and 1932, respectively. This section became more autonomous when it was transformed into the Child and Maternal Department in 1939 as an effort to disseminate the notions of puericulture nationwide. Within this context, the executive branch enacted three decrees related to the HCC in 1937, 1939, and 1943.

Although the contest became official in the mid-1930s, it had already caught the attention of physicians in the early 1920s as a strategy to reduce infant mortality and halt the presumed biological degeneration affecting the population.

As part of the international child welfare campaign that emerged in the 1920s that intertwined socio-medical and educational programs, racial ideologies, hereditary and biological theories, and eugenics for racial betterment, Colombia was not oblivious to this ideological climate. The Colombian government and medical community implemented various medico-social programs and approved legislation in line with the projects discussed in specialized Pan American conferences (Pan American Sanitary Conferences, Pan American Child Congresses etc.). Although child protection was included in programs by several Colombian administrations since the end of the nineteenth century (Vásquez Valencia, 2018, p.110), it became a top priority in the 1920s.

Two factors were responsible for this vivid interest in child protection. First, the state and the medical community became responsible for the welfare of the population, including children; up to that time the Catholic Church had controlled social assistance (see Castro Carvajal, 2007). Second was the new understanding of child mortality as a sociobiological problem attributed to parental ignorance, negligence, social vices, and poverty. In the light of this new interpretation, the new societies to protect children (Bogotá’s Pediatric Society and the La Goutte de Lait program, founded in 1917 and 1919, respectively) worked to reduce child mortality by teaching the principles of puericulture and eugenics to mothers: the purpose was to “develop strong and fit organisms instead of useless rachitic beings who later will be racially-degenerated elements” (Pardo Calderón, 1920, p.19).

The problem gained public attention when a series of talks organized by the Bogotá Student Assembly entitled “Problems of Race in Colombia” featured six Colombian experts to explain the causes of degeneration of the “Colombian race” and the possibilities to reverse this unfortunate process. The speakers described causes ranging from hereditary biological determinism to environmental effects that included government negligence (see López de Mesa, 1920). One, Jorge Bejarano (1888-1961), the cofounder of Bogotá’s Pediatric Society, director of the La Goutte de Lait program from 1919 to 1924, and one of the most respected Colombian hygienists in the country as well as abroad, argued that Colombians were not degenerated. In Bejarano’s opinion, the population was undergoing a process of physical and moral modification that would ultimately improve the country (Bejarano, 1920a, p.191). But even if Colombians were not degenerated, he was convinced that external conditions could negatively impact the population’s mental and physical health. His medical work, focused mainly on public hygiene and child welfare, guided his comments highlighting the “social diseases” that harm children, lead to child delinquency, and finally increase child mortality.

Child mortality was one of Bejarano’s (1920b, p.222) greatest concerns; he alluded to the “maternity prize” as a mechanism to expand and improve the Colombian population. This mention came during his second speech, in which addressed the high mortality rate at the Bogotá Orphanage. He accused the institution of contributing to the “degeneration of population” by letting 250 of 1,000 children die every year. In his opinion, these deaths were the result of appalling hygienic conditions at the facility and care provided by “repulsive and deprived childminders” who looked after children in their homes just to make money (Bejarano, 1920b, p.223). As references in order to increase and “regenerate” the population, Bejarano referred to France, Germany, and the United States, where political and medical authorities had founded institutions and implemented projects to stimulate good childrearing. One such project involved prizes that encouraged mothers to “raise and take care of their children” (p.223).

Luis-Enrique Pardo Calderón, a Colombian doctor who defended his doctoral dissertation, *Considerations on La Goutte de Lait*, in 1920, also mentioned maternal awards in his work. Because child mortality was a sign of racial degeneration, according to Pardo Calderón (1920, p.18), he concluded that: “To fight against the high rate of child mortality in Colombia, it was necessary to found La Goutte de Lait programs, nurseries, maternal awards, protection for pregnant women etc., in the main cities of the Republic.”

The founding of the Colombian HCC was not only a moral obligation in favor of childhood but also a “patriotic” duty. Indeed, a “healthy and well-fed child represents the nation’s [human] capital and fundamental culture whose cornerstone is the race’s good psycho-physical state. The highest human civilizations …, in which the child was at the heart of their public policies, are testament to progress and development” (Otálora, 1937, p.1). This statement highlighting the link between children, race, and nation (three major components in the project of creating a nation-State) came from Benjamín Otálora (physician and vice-director of the Second Division of the NDH) during the second HCC held on October 12, 1937 in several cities across the country (Decreto n.1201…, 1 Sept. 1937).^5^

The NDH was authorized by government decree to organize this event; the decree also urged all welfare institutions under this department to “reinstate the National Healthy Child Contest,” which was to be held every year (Articles 1 and 6). It established three awards according to the age: best breastfed baby (up to two years of age), best preschooler (ages two to six), and best school-aged child (ages six to twelve). While the main target was early childhood, the contest covered all of childhood up to puberty.

The panel of judges, composed of “a local sanitary agent, a doctor from the Child Welfare Service, a legally-authorized doctor, the mayor of the city/town or a legal representative, and a ‘well-regarded lady’ (chosen by the other members of the panel) award the children and their families with 100 Colombian pesos (COP) that should be divided among the three categories” (Articles 3 and 4). The directors of the local child welfare services were tasked with increasing the prize budget to “arouse mothers’ interest in preserving their children’s physical, mental, and moral health” (Article 4).^6^ By awarding the winning families money or property, the government was also trying to build an image of a welfare state that cared for its population, especially children, in order to ensure the future of the nation. In fact, the Child Congresses held from 1916 to 1942, which highlighted an active government role through re-education, healthcare for children, and the creation of child tribunals and other institutions in charge of children’s needs (Guy, 1998, p.274), advised participant countries to provide family allowances or housing benefits not only as a strategy to protect childhood, but also to solidify the image of a protective state with the power to take control of its population (for example, see Antecedentes…, 1925).

On July 28, 1937, NDH director Benigno Velasco Cabrera (1937) published the general rules for the contest. In addition to the specifics established in the decree mentioned above, these regulations described the desired characteristics of the contest participants in detail:

[the child must] a) be enrolled in a ‘Healthy Child Office’ recognized by the national child welfare entities; b) have been exclusively breastfed until the age of six months; c) be vaccinated against smallpox; d) have no signs of hereditary defects; e) have psycho-physical development in line with his/her ‘normal physiological’ development inherent to the age (weight, height, chest circumference, dentition, skull ossification; … f) have no any organic defects; g) and have personal hygienic habits and [dwell] in a hygienic household (Ministerio…, 1939, p.21).

These regulations clearly targeted the type of child desired by the government according to eugenic, hygienic, and sociobiological principles. This selection of children with the desired physical and moral features proved that even if the country’s political and intellectual elites desired to “civilize” the nation via hygiene and education, a hereditary and biological component remained crucial in this undertaking.

The Second Division of the NDH was charged in the 1937 decree with setting the date for the contest: October 12. According to its Vice-Director, Benjamín Otálora, the choice “harmonized symbolically with Columbus Day” (commemorating the Spanish arrival in the Americas in 1492, known as Día de la Raza in Latin America) (Otálora, 1937, p.2). This was a very significant choice from a racial and biological point of view. Some experts believed that the clash between the “races” on October 12, 1492 produced a new type of man that was allegedly “degenerated” due to racial mixing. By selecting this same date for the contest highlighting children of the twentieth century who represented the beginning of a new “regenerated race,” the country started with a clean slate after a long history of degeneration among its population.^7^

On October 12, 1937, the national HCC was held in 33 cities, including Bogotá.^8^ The contest in Bogotá had strong support from Colombia’s First Lady, María Michelsen de López, and the city government, which allocated COP $500 for the awards and other expenses. It was thoroughly covered by the press, especially the national newspaper *El Tiempo*; this is no surprise, since its founder and director Eduardo Santos (future president of Colombia from 1938 to 1942) was a Liberal partisan. In a series of five articles published between October 2 and 12, *El Tiempo* documented the contest process and featured some illustrations that showed not only the physical condition of the winning children, but also the engagement of the executive branch (via the First Lady).

On October 9*, El Tiempo* announced the winners from the final selection that had taken place the day before at the presidential palace. The six children selected in the first eliminatory round represented the six nurseries operating in the capital. Betulia Toledo, an 8-month-old baby, was declared the winner of the contest; according to the article, the girl “met the requirements of a perfect creature” (Betulia..., 9 Oct. 1937, p.14). Her family was given a COP $50 mortgage bond and a certificate. The four runners-up each received a COP $25 mortgage bond, and the official award ceremony was held at the Chapinero nursery on October 12.

Although Otálora reported some difficulties in some cities (without going into detail), he described the contest as exceeding all expectations. This was supported by several articles in *El Tiempo* that reported other “successful experiences” with HCCs held in medium-sized cities like Ibagué (Tolima), Popayán (Cauca), and Tunja (Boyacá). Organized by the Sanitary Unit in each city, these contests were described in the articles as quite successful considering the large number of families, mainly from “humble backgrounds,” who participated in the contest (Con un regio…, 13 Oct. 1937; Con grande… 14 Oct. 1937; El Concurso…, 17 Oct. 1937).

Benjamín Otálora went on to write a report highlighting this unquestionable success, and also noted errors to be corrected in future competitions. First, the private commercial and industrial sector needed to be involved in the national HCC to assist with funding; this would help generate awards that would best meet the needs of the winning families, which included home improvements, sanitary projects, acquisition of animals etc. (Otálora, 1937, p.13), and would also attract peasant as well as urban families. He went on to say that a farm with all the modern sanitary facilities could be given to families with a father working in the countryside, and a small house to working-class families in town (p.13). By engaging the private sector, the “rich,” and the Departmental Assembly, the Second Division could also consider a contest within the department that brought together the winners in each town.^9^ This initiative would catalyze interest in protecting and caring for children, especially in the countryside. The government drive to implement the contest in the countryside was part of its ambition to “sanitize and educate” the rural population in a country that was mostly agrarian in the 1930s, with a lack of proper sanitary facilities and hygienic habits that was seen as an obstacle to modernization (Muñoz Rojas, 2022, p.92; on health programs planned by Liberal governments in rural areas, see Jalil, 2019; Botero-Tovar, 2021). It also would consolidate the idea of a “health” regulation established by the sanitary authority. This kind of program would reinforce medical legitimacy among the population by proving that “science” was working in favor of the society and strengthening Colombia’s national identity.

The Colombian national HCC reached its strongest momentum when the Child and Maternal Department took over its organization. According to Decree 378 of 1939:

The Director of the Department will lead a permanent campaign [in favor of child protection] in the institutions attached to it. The campaign will teach the principles of hygiene and child nutrition, as well as other conditions that may have an impact on the child’s health and welfare. To do so, the Department will carry out an inspection of publications, advertising posters, magazines etc., edited by the Ministry, and will organize permanent and itinerary exhibitions on puericulture, Weeks of the Child, Healthy Child Contests, talks, film advertisements etc. (Decreto n.378..., 1 Mar. 1939).

The director and vice-director of the Child and Maternal Department (Héctor Pedraza and Rubén Gamboa Echandía) lent an eugenic dimension to the contest, like many other socio-medical programs, since it would help to avoid the transmission of hereditary defaults (dysgenic elements) resulting from malnutrition in mothers and children. In a 1940 manual edited by Pedraza and Gamboa Echandía entitled *Integral hygiene and child nutrition*, puericulture was described as an essential element of eugenics in order to stop the passage of hereditary defects to offspring (Gamboa Echandía, Pedraza, as cited in Pohl Valero, 2014, p.474). The first chapter of the manual explained the concepts of eugenics and advocated “eugenic measures” such as “sterilization, combat against racial mixing, regulation of immigration, regulation of marriage, moral and sexual instruction, instruction for children suffering from abnormalities, and social hygiene measures” (p.474).

In 1943, Colombia’s President Darío Echandía and Minister of Labor, Hygiene, and Social Assistance Jorge-Eliécer Gaitán revoked Decree 1201 of 1937, which regulated the HCC. Its replacement, Decree 2341 of 1943, defined that the national winner would be chosen nationwide according to guidelines defined by the ministry (Decreto n.2341…, 7 Dec. 1943). The award – a home – would be given to the mother, childminder, or governess if she could demonstrate that the winning child had been taken to the Healthy Child Office during a two-year period and that the family had followed the hygienic guidelines to beyond reproach; periodic house calls by a visiting nurse or any other sanitary authority would validate that the family indeed met the requirements (Decreto n.2341…, 7 Dec. 1943). The decree also emphasized the importance of teaching the principles of puericulture to mothers as a mechanism to preserve childhood.

## Final considerations

Colombia’s national HCC remained another “prophylactic and educational” mechanism to control social health by interfering in families’ private lives in order to create a national identity and define what Catalina Muñoz has called “Colombianness.” The contest demonstrates the Liberal Republic’s will to “civilize” the population by implementing health programs focusing on children. Instead of replacing the biological approach that prevailed during the first two decades of the twentieth century, this state interventionism came to complement it. The idea of a “degenerated race” from a biological perspective, which could be generated via medical-social programs to protect future generations, gained traction in the 1930s through the rise of eugenics. The fact that the eugenic principles were implemented by means of puericulture revealed that the Colombian experts applied a “preventive” and “persuasive” eugenic modality (even if this movement was not institutionalized through the founding of institutions) that favored the interests of various groups ranging from the most conservative to the most liberal factions, and especially the Catholic church.

From a transnational perspective, the HCC shows that Colombian socio-medical programs were in line with those of other countries and discussed at various specialized meetings including the Pan American Sanitary Conferences, Pan American Child Congresses, and Pan American Conferences of Eugenics and Homiculture. These programs sought to protect childhood and, by extension, protect the biological component of the population which was essential for the future of the nation.

## NOTES

1 The idea quickly expanded across the rest of the country, and the contest was organized in other big cities.

2 To participate, the babies had to be at least one year old and breastfed.

3 The Homiculture League was founded by the Cuban eugenicists Domingo Ramos and Eusebio Hernández-Pérez. According to its proponents, homiculture applied the principles of puericulture not only to children but also adults in order to establish a link between “the good state of the human being in all his dimensions and the capacity of a nation to reach its goals of peace, order, and prosperity” (Stern, 2016, p.7).

4 According to the English historian Nancy Stepan (1991, p.101), Latin American eugenics was “preventive,” since it “treated the highly complex social results of misery and poverty with biological metaphors of heredity and race improvement. The focus on the supposedly permanent degenerations caused in human populations by racial poison led to an emphasis on the need for state programs of sanitary registration and control of the sickly, undernourished, and badly housed poor; to the use of techniques of ‘mental hygiene’ for the eugenization of ‘undisciplined’ workers as a way to achieve their ‘purification;’ to an exhortation to moral virtue as the centrepiece of sanitary education.”

5 In a decree approving Resolution 144 of May 5, 1937, which established the Colombian HCC.

6 The Healthy Child Office was founded as a result of the HCC to disseminate the concepts of puericulture to reduce child mortality and encourage breastfeeding. Breastfeeding was always a contentious issue at the time, not only in Colombia but also at international congresses like the Pan American Child Congresses, since many doctors opposed formula. But at the same time, mother’s milk was at times considered damaging, since the “bad characteristics” of mothers could be passed on to their children.

7 In 1922, the Third Pan American Child Congress suggested that all countries in the region organize “Children’s Day” on October 12 (Terceiro…, 1924, p.138).

8 The cities were located throughout the country: Palmira, Barbacoas, Aguadas (Caldas), Florencia, Tunja, Villavicencio, Coper, Vélez, Socorro, Ocaña, Guapi, Sogamoso, Honda, Valledupar, San Andrés (Santander), Bucaramanga, Popayán, Garzón, Pereira, Tumaco, Líbano, Restrepo, Rionegro (Santander), Quibdó, La Dorada, Girardot, Armenia, Abejorral, Chiquinquirá, Bogotá, Cúcuta, and Ibagué.

9 At the time, Colombia was divided into 24 administrative areas: 15 *departamentos,* two *intendencias*, and seven *comisarias*, which were further subdivided into cities and towns. Today, there are 32 *departamentos*.
